# Brexpiprazole—Pharmacologic Properties and Use in Schizophrenia and Mood Disorders

**DOI:** 10.3390/brainsci13030397

**Published:** 2023-02-25

**Authors:** Marcin Siwek, Krzysztof Wojtasik-Bakalarz, Anna Julia Krupa, Adrian Andrzej Chrobak

**Affiliations:** 1 Department of Affective Disorders, Jagiellonian University Medical College, Kopernika St. 21a, 31-501 Cracow, Poland; 2 Department of Psychiatry, Jagiellonian University Medical College, Kopernika St. 21a, 31-501 Cracow, Poland; 3 Department of Adult Psychiatry, Jagiellonian University Medical College, Kopernika St. 21a, 31-501 Cracow, Poland

**Keywords:** antipsychotic drugs, brexipiprazole, adverse effects

## Abstract

In 2002, the first III generation antipsychotic drug was registered—aripiprazole. Its partial dopaminergic agonism underlies its unique mechanism of action and the potentially beneficial influence on the positive, negative, or cognitive symptoms. Due to its relatively high intrinsic activity, the drug could often cause agitation, anxiety, or akathisia. For this reason, efforts were made to develop a drug which would retain the positive favorable actions of aripiprazole but present a more advantageous clinical profile. This turned out to be brexpiprazole, which was registered in 2015. Its pharmacodynamic and pharmacokinetic profile (similarly to the other most recent antipsychotics, i.e., lurasidone or cariprazine) shows promise of increasing the effectiveness of schizophrenia treatment in the dimensions in which the previous antipsychotics were not sufficiently effective, including negative, depressive, or cognitive symptoms. Like other new antipsychotics, it can also be useful in the treatment of mood disorders, for instance drug-resistant depression. Previous reviews focused on the use of brexpiprazole in specific diagnostic groups. The aim of this article is to provide the readers with an overview of data on the mechanism of action, clinical effectiveness in all studied diagnostic groups, as well as potential drug–food interactions, and the safety of brexpiprazole.

## 1. Pharmacological Profile of Brexipiprazole

### 1.1. Structure and Class

**Figure 1:**
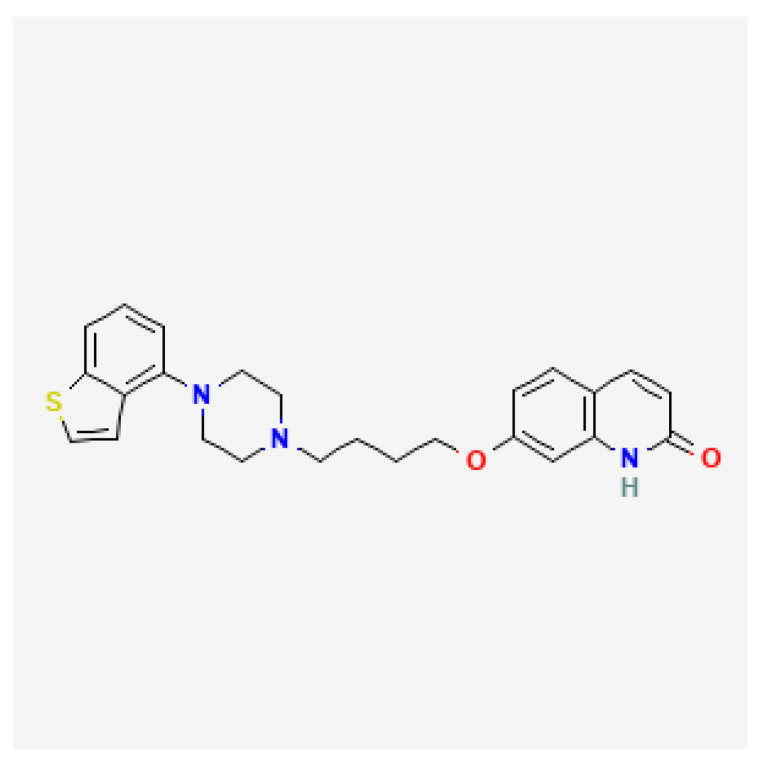
Brexpiprazole chemical structure based on [1].

Brexpiprazole is a quinol derivative, its structure very much resembles that of aripiprazole (Figure 1) [1]. Compared to aripiprazole, brexpiprazole has an additional thiophene ring. This different structure contributes to dissimilar pharmacodynamic properties. As opposed to aripiprazole, brexpiprazole (1) has less intrinsic D2 activity which translates to lower risk of akathisia and EPS, (2) presents higher potency at 5HT1_a_ which may result in better antidepressant, anxiolytic, and procognitive effectiveness, (3) shows higher potency at 5HT2_a_ and therefore is less likely to cause akathisia and insomnia. According to the neuroscience-based Nomenclature 2 classification, it is a partial dopamine and serotonin agonist (D_2_, 5HT_1a_) as well as a serotonin antagonist (5HT_2a_) [2,3].

### 1.2. Pharmacodynamics

In in vitro studies, brexpiprazole acts as a partial agonist of 5HT_1a_, D_2_, and D_3_ receptors [4]. Its impact on 5HT_1a_ receptors might translate into precognitive and mood-enhancing effects as well as a reduced risk of extrapyramidal symptoms (EPS). The partial agonism of D_2_ receptors accounts for the antipsychotic action of the drug, it also mandates that the risk of EPS and hyperprolactinemia (hPRL) is lower compared to antipsychotics which display the complete antagonism of D_2_. Regarding the D_2_ receptors, brexpiprazole demonstrates a level of intrinsic activity that is comparable to cariprazine or aripiprazole, meaning that it is for the most part antagonistic. Therefore, compared to aripiprazole, it might present a lower occurrence of adverse effects (AE) such as akathisia, nausea, or vomiting [5]. In the therapeutic dose range, brexpiprazole occupies 59–75% of dopaminergic receptors, approximately as much as the drugs from older generations of antipsychotics, which warrants good effectiveness on positive psychotic symptoms [6]. The partial agonism of D_3_ might result in increased effectiveness on negative symptoms of schizophrenia as well as recognitive and antidepressant potential [5]. The drug also acts as an antagonist on 5HT_2a_, 5HT_2c_, 5HT_7_, alfa-_1_, and H_1_ receptors. The antagonism on 5HT_1a_ might cause sedative and anxiolytic activity, increased antipsychotic effect, and decreased risk of EPS and hPRL. The antagonism of 5HT_7_ translates to procognitive and antidepressant effect, especially in combination with the partial agonism of 5HT_1a_, and it might also have a positive impact on the negative schizophrenia symptoms. Although low, the antagonism of 5HT_2c_ might produce antidepressant action but also increased appetite. The moderate antagonism of α_1_ might cause more sedation, higher risk of orthostatic hypotension, and impact the effectiveness of the drug in post-traumatic stress treatment, in particular nightmares (which was addressed in preliminary reports regarding this issue). The α_2_ antagonism might influence the antipsychotic action of the drug, however the H_1_ antagonism increases the risk of weight gain and sedation, and it also results in an anxiolytic and tranquilizing effect [7,8,9,10]. The in vitro studies in animal cell lines also indicated the inhibitory activity on monoamine, 5HT, NA, and DA transporters, which was comparable to that of serotonin and noradrenalin reuptake inhibitors (SNRI); however, studies on human cell lines reported only a small impact on these transporters. Additionally, these results were not replicated in other in vitro studies on animals [11]. The comparison of affinities of brexpiprazole and other partial agonists of D_2_ for specific receptors is presented in Table 1.

### 1.3. Pharmacokinetics

After oral ingestion, brexpiprazole is easily absorbed, it is characterized by a high 95% bioavailability. Food has no significant effect on the pharmacokinetics of brexpiprazole, therefore it might be taken with or without a meal which is more convenient compared to lurasidone which has to be taken with a meal of at least 350 kcal or ziprasidone which should be taken with a meal of at least 500 kcal, and potentially increases the chance of good adherence. What is more, the time from the last meal or to the next meal has no influence on brexpiprazole absorption, which enhances the adherence compared to, i.e., quetiapine XR, which should be taken at least an hour before the next meal or sulpiride which should be taken either one hour before the meal or two hours after it [14]. The volume of distribution after intravenous administration is approximately 1.56 ± 0.42 L/kg, which suggests an extravascular distribution. The drug has significant lipophilic properties, which enables the blood–brain barrier transfer, but could also result in storage in fat tissue [15]. Maximum drug concentration is reached 4 h after oral ingestion, the biological half-life is 91 h [16]. Because the drug’s half-life is long, an omission of a dose has less of an impact on its effectiveness vs. other antipsychotics with shorter half-lives. A study comparing the risk of relapse after antipsychotic discontinuation indicated that 4 weeks after the brexpiprazole was stopped only 8% of patients reported the recurrence of disease. After this time, the drug–placebo separation was noted, which was a second-best result following the one for cariprazine relapse of 5% after 4 weeks, with drug–placebo separation after 6.6 weeks. For the other antipsychotics, the results ranged between 13 and 34% for the risk of relapse after 4 weeks and between 1.1 and 2.7 weeks for the drug–placebo separation [17]. Brexpiprazole is metabolized by cytochrome enzymes CYP2D6 and CYP3A4. Its metabolism produces an active metabolite DM-3411, which has a pharmacodynamic profile similar to the parent drug but is less potent and has a lower penetration to the central nervous system and therefore has no significant impact on the therapeutic effect. Both the parent drug and the active metabolite to a great extent (99%) bind to the serum proteins. Therefore, both brexpiprazole and DM-3411 could potentially compete with other drugs which bind to serum proteins which may potentially result in (1) increased levels of free form of the concomitant drug should it bind to serum proteins with less potency and higher risk of its adverse effects, (2) increased levels of free brexpiprazole and free DM-3411 should the concomitant drug bind to serum proteins with higher potency and higher effects as well as risk of adverse effects of brexpiprazole/DM-3411 despite the relatively low dosing. Nonetheless, as reported by the producer, the results of in vitro studied indicated that protein binding of brexpiprazole is not influenced by warfarin, digoxin, or diazepam [18]. After repeated doses, the steady state is reached in 10–12 days. Brexpiprazole is mainly eliminated with urine and feces, the unmetabolized drug accounts for 14% in the first and <1% in the second case [19]. The therapeutic concentration of brexpiprazole ranges between 40–140 ng/L and the alert level 280 ng/L. No standards advise routine plasma drug concentration monitoring; however, it could be clinically useful [20]. No significant differences were reported regarding the pharmacokinetics in patients 65 y or older. Subjects with moderate or severe liver or kidney damage are at risk of increased drug exposition, therefore the dose should be lower. Slow CYP2D6 metabolizers show about 30% lower drug clearance and are at risk of increased drug exposure, same as patients taking CYP2D6 inhibitors. In these circumstances, the Summary of Product Characteristics (SmPC) advises dose modification: a dose reduction by 50%. However, the physiological modeling of drug pharmacokinetics indicated that this might delay the occurrence of therapeutic effects and prolong the treatment. The authors of the abovementioned study suggest that the recommended dose should be administered two times a day (b.i.d) in the first week of treatment [21]. Subjects who simultaneously receive CYP3A4-inhibiting drugs are also at risk of higher brexpiprazole exposition.

Furthermore, drug and nutrient interactions need to be taken into account. Recently, a study on rats suggested that concurrent administration of brexpiprazole and grapefruit and pomegranate juices (CYP3A4 inhibitors) increased systemic exposure to brexpiprazole [22]. Several drugs (i.e., azoles, foods, and drinks) might inhibit CYP3A4, that is, red wine, beer, grapefruit, pomegranate, cranberry, lime, pomelo, onion and tomato juice, parsley, thyme, celeriac, legumes, garlic, licorice root, Seville orange products (juice, jam, marmalade), black/long pepper, and saffron. Some nutritive products may also inhibit CYP2D6, that is, Glycyrrhiza inflata liquorice root products (spice, confectionery, tea, sambuca), black/long/chili pepper, and saffron. Consequently, patients should be informed of the potential interactions with both drugs and foods and drinks. On the other hand, the risk of drug–drug or drug–nutrient interactions applies to the majority of antipsychotics (aside from amisuplride and sulpiride which are not metabolized by CYP 450 enzymes). Furthermore, the risk of drug–nutrient interactions is in most cases minimal and reaches the level of clinical significance only in patients who consume substantial amounts of the abovementioned products (i.e., due to consumption of the dietary supplements) [14]. Concurrent administration of CYP3A4 inductors could decrease the effectiveness of the drug. It should be noted that aside from drugs which are CYP3A4 inductors (i.e., carbamazepine, glucocorticosteroids), some foods (ginger) might also include CYP3A4 [14]. While in vitro studies indicate that brexpiprazole might act as a weak inhibitor of CYP2B6, 2D6, and 3A4, this effect seems to have no clinical impact on the risk of drug interactions, as no reports of studies or cases suggesting its significance are available [23]. Moreover, the product monograph states that oral brexpiprazole had no effect on the metabolism of single doses of dextromethorphan (a CYP2D6 substrate), lovastatin (a CYP3A4 substrate), or bupropion (a CYP2B6 substrate), and that brexpiprazole did not affect the absorption of single doses of drugs that are substrates of BCRP transporter (rosuvastatin) and PgP (P-glycoprotein) transporter (fexofenadine). The producer states that no dosage adjustment in needed in the case of CYP2D6, CYP3A4, CYP2B6, BCRP, and PgP substrates during concomitant administration with brexpiprazole [18]. The physical modeling of the drug’s pharmacokinetics suggests a potentially significant impact that obesity might have on the biological half-life of the drug and the time in which the steady state is reached, which in this study was extended by approximately 9 days, probably due to the high lipophilicity of brexpiprazole and an almost three times higher volume of distribution observed in patients with BMI > 35. Hence, the authors of the mentioned study suggest an algorithm alternative to SmPC in the first phase of the treatment—1 mg b.i.d. in days 1–4, 2 mg b.i.d. in days 5–7, and 4 mg once a day (q.d.) in the next days. For the obese patients who are also slow CYP2D6 metabolizers, they propose the following dosing regimen: 0.5 mg b.i.d. in days 1–4, 1 mg b.i.d. in days 5–7, 2 mg b.i.d. in days 8–18, and 2 mg q.d. in the next days [15].

## 2. Clinical Use

### 2.1. Schizophrenia—Acute Episode (up to 6 Weeks)

Short-term studies of brexpiprazole monotherapy indicate its effectiveness in reducing the positive, negative, and cognitive symptoms, severity of aggression, as well as the levels of improvement and functional remission. The number needed to treat (NNT) for treatment response in acute schizophrenia of brexpiprazole is 7, which is lower than observed in the case of cariprazine treatment (NNT 10) and higher than that reported for lurasidone treatment (NNT 4–7 depending on the dose of the drug) [24]. A meta-analysis of studies exploring the links between the effectiveness and drug dose showed that, regarding the negative symptoms, the dose of 2 mg/d resulted in the maximal effect and then a plateau was observed. Concerning the positive symptoms, the effective dose (ED95) was 4 mg/d, however the curve was upward, suggesting a potential increase in effectiveness in doses above 4 mg/d [25]. The studies on acute schizophrenia episode treatment are presented in Table 2. 

### 2.2. Schizophrenia—Long-Term Treatment

Available randomized controlled trials (RCTs) and open-label studies indicate that the therapeutic effects observed in short-term studies are either sustained or increase with the treatment time. Additionally, brexpiprazole use in monotherapy provides a good chance of achieving improvement or functional remission. The worse the pretreatment functioning of the patient, the higher the probability of improvement; on the other hand, the better the initial level of functioning, the higher the likelihood of remission. Brexpiprazole treatment is also associated with a lower risk of relapse and a longer time to treatment discontinuation compared to other antipsychotics. The NNT for the relapse prevention of brexpiprazole is 4, which is similar to the ones reported for aripiprazole (NNT 5), cariprazine (NNT 5), or olanzapine (NNT 3) [39]. Studies on long-term schizophrenia treatment are displayed in Table 3.

### 2.3. Treatment-Resistant Depression (TRD)

The effectiveness of brexpiprazole was extensively studied in augmenting the antidepressant treatment in TRD and its effects were noted in reducing the symptoms in all domains of depression. It improved the patients’ functioning and showed effects in patients with coexisting anxiety or anger. In previous studies it was also effective in the treatment of patients who had no treatment response to other therapies. NNT for treatment response in TRD of adjunctive brexpiprazole is 12, which is similar to that observed in the case of treatment augmented with quetiapine (NNT 12) and higher than that reported for adjunctive aripiprazole (NNT 9), risperidone (NNT 6), or the combination of olanzapine and fluoxetine (NNT 6) [45,46]. The therapeutic effects might be noted within a week of treatment. Studies assessing brexpiprazole as an adjunctive drug in TRD are presented in Table 4.

### 2.4. Bipolar Disorder

Preliminary studies suggest the potential effectiveness of brexpiprazole as monotherapy or adjunctive drug in the treatment of bipolar depression. While pilot studies were conducted in small groups of patients, their results in TRD and the profile of action raise hope that further studies will confirm these preliminary data. On the other hand, no effectiveness of brexpiprazole monotherapy on manic episodes was shown. The studies of brexpiprazole effectiveness in bipolar disorders are summed up in Table 5.

## 3. Safety and Tolerance

The existing RCTs and open-label studies indicate the good safety and tolerance of brexpiprazole. The majority of AEs are mild or moderate, while severe AE are rare. The risk of discontinuation due to AEs is comparable to these observed for other new antipsychotics. The studies on safety are summed up in Table 6.

### 3.1. Weight Increase and Metabolic Syndrome

Due to the relatively high affinity for the H_1_ receptor, brexpiprazole causes the weight increase more often than other III generation neuroleptics. In a meta-analysis of clinical studies it was noted that for the treatment with doses 2–4 mg/d, the mean weight gain was 0.95 kg and the number needed to harm (NNH) was 20 (for comparison, NNT for cariprazine was 50 and mean weight increase 0.55, for quetiapine NNH was 34 and mean weight gain 0.99 kg, for risperidone NNH was 12 and mean weight increase was 1.5 kg) [62]. Despite the significant risk of weight gain, no increase in the risk of metabolic syndrome was observed during brexpiprazole treatment. Moreover, in a study on 37 subjects, it was reported that a switch of antipsychotic (from risperidone, olanzapine, blonanserine, haloperidole, aripiprazole, paliperidone, and perospirone) to brexpiprazole was associated with the decrease in weight and improvement of metabolic parameters [63]. In a different study on 186 participants, who were switched from the previous neuroleptic to brexpiprazole, no changes in lipid profile and a slight increase in weight were noted in those initially treated with antipsychotics other than aripiprazole. The patients who were previously treated with aripiprazole had a mean increase in weight of 1.1 kg [64].

### 3.2. EPS

The most common EPS observed during brexpiprazole therapy is akathisia, which is significantly less common while using brexpiprazole vs. while using other new neuroleptics such as cariprazine, lurasidone, or aripiprazole. The low risk of EPS/akathisia due to brexpiprazole treatment could be explained by its relatively low (lower than this reported for aripiprazole) intrinsic D2 activity. Indeed, previous studies showed that regarding the risk of akathisia the NNH of brexpiprazole ranged from 15 (in depressed patients) to 112 (in patients with schizophrenia). The NNH for akathisia of aripiprazole ranged from 12 to 30 (in depressed patients) and 6 to 12 (in schizophrenia patients, depending on the dose of the drug). The NNH for EPS/akathisia of aripiprazole was 4 and NNH for akathisia in the case of cariprazine ranged from 7 (in patients with manic or mixed episodes) to 12 to 20 (in patients with schizophrenia) depending on the dose of the drug [45,46]. In the previously mentioned studies, a change from brexpiprazole to a different antipsychotic either did not produce an exacerbation of EPS [64] or resulted in an improvement of EPS [63].

### 3.3. hPRL and Sexual Functions

In the short-term studies, AEs linked to prolactin levels were noted in 1.8% of patients on brexpiprazole compared to 0.6% of those receiving placebo, while in the long-term studies AEs were noted in 1.7% of subjects treated with brexpiprazole. hPRL exceeding >3x the upper the limit of normal were noted in 1.5% of women and 1.6% of men using brexpiprazole and 3.6% of women and 3.4% of men receiving placebo. In the long-term observations, similar hPRL increases were noted in 5.3% of women and 2.0% of men [65]. Both in the short- and long-term studies an improvement of sexual functioning was noted in patients with TRD treated with brexpiprazole [66].

### 3.4. Other AE

Of studies on large patient groups, none reported a prolonged QTc interval or hematological AE, one case of seizure was noted. In a post hoc study on a group of 410 subjects, a link between prolonged QTc interval and brexpiprazole use was observed, however only five of all patients in this study received this drug [67]. No cases of significant hepatotoxic reactions were observed during the treatment.

The data on AE of brexpiprazole vs. aripiprazole and cariprazine are presented in Table 7 [7,68].

In Table 8 values of NNH for the most important AE of brexpiprazole, cariprazine, lurasidone, and aripiprazole are summed up [69,70].

### 3.5. Gaps in Knowledge Regarding Brexpiprazole

Little is known of the effectiveness of brexpiprazole in schizoaffective disorder. Only two of the conduced studies of brexpiprazole in schizophrenia included patients with schizoaffective disorder, however they constituted less than 10% of the subjects [63,71]. Considering the pharmacological profile of brexpiprazole and its effectiveness in schizophrenia, major depression, and probably also bipolar depression, the drug could potentially be effective in schizoaffective disorder. This issue needs to be addressed in future studies. As mentioned earlier, the studies assessing the effectiveness of brexpiprazole in bipolar depression are preliminary and further research on this issue is necessary.

## 4. Conclusions

Brexpiprazole is an antipsychotic showing effectiveness in both short- and long-term treatment of schizophrenia and TRD. It is also effective in bipolar depression as well as agitation and aggression related to the course of dementia. It has an advantageous pharmacokinetic profile which decreases the risk associated with a dose omission and a pharmacodynamic profile which ensures the low occurrence of AEs, good tolerance, and effectiveness in reducing the positive symptoms of psychosis as well as affective and cognitive symptoms.

## Figures and Tables

**Table 1:** The comparison of affinities of brexpiprazole and other partial agonists of D_2_ for specific receptors. Additionally, clinical effects of agonism and antagonism of those receptors has been summarized based on [7,12,13]—modified.

	Effects of Receptors Agonism/Partrial Agonists	Effects of Receptors Antagonism, Reversed Agonism	Brexpiprazole	Aripiprazole	Cariprazine
D_2_	Partial agonism: reduction in EPS and hPRL, anticonvulsive effect.	Antipsychotic and antimanic effect, decrease in agitation and aggressive behaviors. Increase in EPS, PRL levels, cognitive impairements, severity of negative symptoms. Proconvulcive effect and possible weight gain.	0.30 #	0.34 #	0.49 #
D_3_	Reduction of negative symptoms.	Possible antipsychotic, antidepressant, procognitive effect.	1.1 #	0.8 #	0.085 #
5HT_1a_	Antidepressant, anxiolytic, and procognitive effect. Reduction in EPS and sexual impairements associated with serotonin reuptake inhibition.	?	0.12 #	1.7 #	2.6 #
5HT_2a_	Propsychotic and possible antipsychotic effects. Increased severity of impulsive behaviours, insomnia, neuroplasticity, and prolactive release. The risk of inducing serotonin syndrome.	Antipsychotic, anxiolytic, and non-rapid eye movement sleep-promoting effect. Reduction of PRL, EPS, and impulsive behaviours. Potentialization of the serotonin reuptake inhibition effects. Increased sedation and insulin resistance.	0.47 *	3.4 *	18.8 *
5HT_2c_	Anorectic effect. Reduction of impulsive behaviours.	Procognitive and antidepressant effects. Increased weight gain.	34 *	15 #	134 *
5HT_7_	?	Anidepressant, antipsychotic, and procognitivive effect. Decreased severity of negative sumptoms and duration of rapid eye movement sleep.	3.7 *	29 #	111 *
α_1_	Hypertension	Hypotonia (including orthostatic hypotonia), tachycardia, nasal swelling, ejaculation impairements, sedation, weight gain, increased risk of metabolic syndrome, priapism, urinary incontinance, proconvulsive effect.	3.8 *	57 *	6.88 *
α_2_	Procognitive and analgesic effect. Increased sedation, hypotonia. Pro- or anticonvulsive effect dependent on the drug dose.	Antidepressant and possible procognitive effect. Increased nightmares.	0.59 *	37.9 *	?
	?	Sedation, weight gain. Possible proconvulsive effect.	19 *	61 *	23.2 *
M	Procognitive proconvulsive and possible antipsychotic effect. Increased EPS. Possible sialorhea.	Reduction of EPS. Increased cognitive impairement, agitation, possible propsychotic effect. Peripheral anticholinergic symptoms.	>1000	>1000	>1000

Antagonism—*, partial agonism—#, effect unknown—?. The values presented in the table are values of the dissociation constant Ki (nM); the lower the value, the higher the affinity for the receptor. EPS—extrapyramidal symptoms. PRL—prolactine.

**Table 2:** The studies on acute schizophrenia episode treatment with the use of brexipiprazole.

Study	Methodology	Results
RCT, Correl et al. [26]	N = 459; SZ; BRX * 0.25 mg, 1 ± 0.5 mg, 2.5 ± 0.5 mg, 5 ± 1 mg vs. ARI 15 ± 5 mg vs. placebo	ARI and BRX > placebo; p = ns
RCT Beacon, Kane et al. [27]	N = 674; SZ; BRX * 1 mg, 2 mg and 4 mg vs. placebo	PANSS: BRX 4 mg > placebo, p = s; BRX 1 mg and 2 mg > placebo, p = ns
RCT Vector, Correl et al. [28]	N = 636; SZ; BRX * 0.25 mg, 2 mg and 4 mg vs. placebo	PANSS: BRX 2 and 4 mg > placebo (total score, agitation, positive and negative symptoms subscales), p = s; CGI-I and CGI-S: BRX 2 mg and 4 mg > placebo, p = s PSP: BRX 2 mg > placebo, p = s; BRX 0.25 mg = placebo
RCT Lighthouse, Marder et al. [29]	N = 468; SZ; BRX * 3 mg and 4 mg vs. QUE XR 600 mg and 800 mg vs. placebo	PANSS: BRX > placebo, p = ns; QUE > placebo, p = s; BRX and QUE > placebo in a post hoc analysis of covariance
RCT, Ishigooka et al. [30]	N = 459; SZ; BRX * 1 mg, 2 mg and 4 mg vs. placebo	PANSS: BRX 2 mg > placebo, p = s; disorganized thinking: BRX 2 mg > placebo, p = s; negative symptoms BRX 1 mg > placebo, p = s BRX 4 mg > placebo, p = ns
RCT, Citrome et al. [31]	N = 97; SZ; BRX * 3–4 mg vs. ARI 10–20 mg	PANSS, CGI-S: ▼ BRX > ▼ ARI; SLOF: ▼ BRX > ▼ ARI; BIS-11; ▼ BRX > ▲ ARI; n-back: ▲ BRX > ▼ ARI
RCT, van Erp et al. [32]	N = 38; SZ; BRX * 2 mg vs. BRX 4 mg	BIS-11: BRX 0; ▼ activity VLPFC in fMRI BRX 4 mg > 2 mg
Post hoc analysis, Correl, Meade et al. [33,34]	N = 952; SZ; BRX * vs. placebo	PSP (total score and specific domains): BRX > placebo (functional improvement NNT 9; better functioning at the outset NNT 17 vs. 8); functional remission 11% BRX vs. 6.7% placebo (NNT 24); size of improvement and occurrence of treatment response ~severity of symptoms at the outset measured in PANSS
Post hoc analysis, Citrome et al. [35]	N = 1094; SZ; BRX * 2 mg and 4 mg vs. placebo	PANSS, P7 ≥ 3: BRX 4 mg > placebo, p =s, BRX 2 mg > placebo, p = ns; PANSS, P7: BRX 2 mg and 4 mg > placebo; regardless of the positive symptoms and akathisia
Meta-analysis, Reyad et al. [36]	SZ and TRD; BRX * vs. placebo	BRX > placebo in PANSS, CGI-I and PSP; BRX2 mg = BRX4 mg
Meta-analysis, Zhou et al. [37]	N = 2178; SZ; BRX * < 2 mg vs. placebo	BRX < 2 mg = placebo; BRX < 2 mg < BRX 2–4 mg
Network meta-analysis, Kishi et al. [38]	N = 3740; BRX * 2–4 mg vs. ARI 10–30 mg vs. placebo	Treatment response: BRX > ARI and placebo

N—number of participants, NNT—number needed to treat, RCT—randomized controlled trial, SZ—schizophrenia, BRX—brexpiprazole, ARI—aripiprazole, QUE—quetiapine, PANSS—Positive And Negative Symptoms Scale, CGI—Clinical Global Impression, BIS-11—Barratt Impulsiveness Scale. p = s—statistically significant result, p = ns—statistically non-significant result, TRD—treatment-resistant depression, *—monotherapy. ▲—increased score, ▼—decreased score, 0—no change.

**Table 3:** Studies on long-term schizophrenia treatment with the use of brexipiprazole.

Study	Methodology	Results
III-phase RCT Equator, Fleischihacker et al. [40] and its post hoc analysis, Correl et al. [33]	N stabilization = 524 N observation = 202 SZ 1–4 treatment weeks; 12 stabilization weeks; 52 observations weeks: discontinuation before the planned end of the study BRX * 1–4 mg in the acute treatment and stabilization; next BRX vs. placebo	Stabilization phase: ▲ PANSS, GAF and PSP Observation phase: relapse BRX 13 vs. 40 placebo (OR 0.29); time to discontinuation 169 vs. 111 (BRX vs. placebo); PANSS: placebo ▼ BRX 0 GAF, PSP, cognitive functions BRX > placebo Post hoc: functional improvement 70.5% BRX vs. 49% placebo (NNT 5), functional remission BRX 63.9% vs. 55% placebo (NNT 6)
Open-label, Ishigooka et al. [41] and its post hoc analysis, Inada et al. [42]	N = 279; SZ; 98 treatment continuation; 183 de novo; 52 weeks.; BRX * 1–4 mg	PANSS: BRX continuation 0, de novo ▼. Post hoc: <65 y.o. improvement 4–6 week vs. 4–12 > 65 y.o. faster improvement in participants <65 y.o. vs. >65 y.o. (4–6 week vs. 4–12 week); response: 38.9% vs. 28.6%, size of the response: −13.8 vs. −9.0 w PANSS (< 65 y.o. vs. >65 y.o.)
Open-label, Forbes et al. [43] and its post hoc analyses [33,34]	N = 1044; SZ; continuation and de novo; 52 weeks.; BRX * 1–4 mg	PANSS: ▼ BRX continuation and de novo; 34.3% treatment response, 4.2% inefficacy, 10.5% relapse and hospitalization Post hoc: ▲ PSP 80.9–86.2% functional improvement (more often participants with worse initial functioning); 40.4–44.1% functional remission (more often participants with better initial functioning); ▼ hostility weeks 6–10, then continuation
restrospective cohort study, Hishimoto et al. [44]	N = 5260; SZ; 180 days BRX * vs. OLA/QUE/ARI/RIS	BRX 49.1% discontinued treatment, t = 57.7 ± 46.6 days; other antipsychotics 55.2%, t = 51.9 ± 43.3 days; OR BRX 0.86

N—number of participants, NNT—number needed to treat, SZ—schizophrenia, BRX—brexpiprazole, QUE—quetiapine, OLA—olanzapine, RIS—risperidone, PANSS—Positive And Negative Symptoms Scale, CGI—Clinical Global Impression, BIS-11—Barratt Impulsiveness Scale. p = s—statistically significant result, p = ns—statistically non-significant result, PSP—Personal and Social Performance, GAF—Global Assessment of Functioning, *—monotherapy, ▲—increased score, ▼—decreased score.

**Table 4:** Studies assessing effects of the brexpiprazole in treatment-resistant depression.

Study	Methodology	Results
RCT Pyxis, Thase et al. [47]	N = 379; TRD; 6 weeks; BRX * 2 mg vs. placebo	▼ MADRS, from the 1st week on, SDS (work/school, social/family life), CGI-I and HDRS-17; treatment response in MADRS 23.5% vs. 14.7%, in CGI-I 44.4% vs. 27.7%
RCT Polaris, Thase et al. [48]	N = 677; TRD; 6 weeks; BRX * 1 mg or 3 mg vs. placebo	▼ MADRS BRX 3 mg > placebo ▼ SDS BRX 1 and 3 mg > placebo; treatment response MADRS and CGI-I BRX 1 and 3 mg > placebo; BRX 3 mg > BRX 1 mg
RCT Sirius, Hobart et al. [49]	N = 392, TRD; 6 weeks; BRX * 2 mg vs. placebo	▼ MADRS BRX > placebo; BRX > placebo in anxious distress or with previous improvement <25%
RCT Delphinus, Hobart et al. [50]	N = 501; TRD; 6 weeks; BRX * 2–3 mg vs. QUE XR 100–300 mg vs. placebo	MADRS: BRX > placebo, QUE > placebo, p = ns; SDS: BRX > QUE and placebo, p = ns (p = s for family/social life); CGI-S BRX > placebo, QUE = placebo; treatment response BRX > placebo, p = ns
RCT, Bauer et al. [51]	N = 1986, TRD 8 weeks + 24 weeks BRX * 1–3 mg vs. placebo	BRX = placebo
Post hoc analysis, Hobart et al. [52]	N = 2066; TRD; 6 weeks; BRX * 1–3 mg, 2 mg and 3 mg vs. placebo	▼ SDS: BRX > placebo; subscales: ▼ social/family life and school/work
Post hoc analysis, Katzman et al. [53]	TRD; 6 weeks; BRX * vs. placebo	▼ depression symptoms—core (ES 0.36), dysphoria (0.27), psychomotor retardation (0.32), vegetative (0.29), anhedonia (0.43), and fatigue (0.33)
Post hoc analysis, Thase et al. [54]	N = 678; TRD and anxiety 6 weeks BRX * 1 mg, 2 mg or 3 mg	BRX 2 and 3 mg ▼ MADRS with concomitant anxiety, effects from the 1st week on
Post hoc analysis, Nelson et al. [55]	N = 1056, TRD; 6 weeks; BRX * vs. placebo	BRX > placebo in core depression symptoms in MADRS after 6 weeks; improvement after 2 weeks apart from pessimistic thoughts
Open-label Orion, Hobart et al. [56]	N = 2944; TRD; Polaris, Pyxis and Delphinus; 52 weeks, shortened to 26 weeks; BRX * 0.5 mg–3 mg	▼ CGI-S and SDS after 26 and 52 weeks
Open-label, Fava et al. [57]	N = 50; TRD; 6 weeks; BRX * 1–3 mg	▼ anger in KSQ and irritability in IDS-C30; no further bouts of anger in 15 of 17 subjects; ▼ MADRS, CGI-I, CGI-S, exacerbation after discontinuation
Open-label, Davis et al. [58]	N = 37, TRD; 6 weeks; BRX * 2 mg	▼ HAM-A from the 1st week on ▼ anxiety and anger/hostility in KSQ; ▼ MADRS, CGI-S and SDS

TRD—treatment-resistant depression (at least 1 failed course of antidepressant drug), SDS—Sheehan Disability Scale, MADRS—Montgomery Asberg Depression Rating Scale, HAM-A—Hamilton Anxiety Rating Scale, KSQ—Kellner Symptom Questionaire, ES—effect size, *—as an adjunctive drug, ▼—decreased score.

**Table 5:** The studies of brexpiprazole effectiveness in bipolar disorders.

Study	Methodology	Results
Open-lable, Brown et al. [59]	N = 19; BD I and II D; 8 weeks BRX *	▼ MADRS and IDS-SR30 after 4 and 8 and improvement of the quality of life; 68.4% treatment response after 4 weeks, 73.6% after 8 weeks
2 RCT, Vieta et al. [60]	N = 322, 333; N = 381; BD I M; 3 weeks; 26 week extension; BRX ** 2–4 mg	BRX = placebo

BD—bipolar disorder, D—depression, M—mania, IDS-SR30—Self-rated inventory of Depressive Symptomatology, MADRS—Montgomery Asberg Depression Rating Scale, BRX—brexpiprazole, *—monotherapy or adjunctive drug, **—monotherapy, ▼—decreased score.

**Table 6:** The studies on brexipiprazole treatment safety and tolerance.

Study	Methodology	Results
RTC analysis, Kane et al. [27]	SZ, N = 1769, BRX 2–4 mg 6 weeks;	TAE 59.2% SAE 2.3%, discontinuation due to AE 7.4%; NNH akathisia 84, somnolence 60, weight increase 40. No TAE with an incidence of ≥5% and twice that of placebo in patients treated with BRX
Open-label, Forbes et al. [43]	SZ, N= 1031 BRX 1–4 mg 52 weeks	TAE 60.4%, SAE 8%, discontinuation due to AE 14,6%; 18,6% weight increase >7%, on average 2.1 kg, 9.2% weight decrease >7%, 11.6% SZ exacerbation, 8.6% insomnia, 6.4% headache, 1 seizure linked to treatment
Open-label, Hobart et al. [56]	TRD, N = 2938 BRX * 0.5–3 mg 52 weeks	TAE 72.3%, SAE 7.3%, discontinuation due to AE 8.6%; weight increase >7% 17.7%, on average 3.2 kg, somnolence 8%, headache 7.2%, akathisia 6.7%, increased appetite 6.3%, fatigue 6.1%, nasopharyngitis 5.4%, anxiety 5.2%; 1 completed suicide potentially linked to treatment
Open-label, Lepola et al. [61]	TRD, >65 y.o., N = 132 BRX * 0.5–2 mg 26 weeks	TAE 77.3%, SAE 8.3%, discontinuation due to AE 18.9%; fatigue 15.2%, restlessness 12.9%, increased appetite 9.8%, akathisia 8.3%, weight increase 8.3%, anxiety 7.6%, tremor 6.8%, insomnia 6.1%, nasopharyngitis 6.1%, headache 5.3%

AE—adverse effect, TAE—treatment-emergent adverse effect, TRD—treatment-resistant depression, SAE—severe adverse effect; SZ—schizophrenia, BRX—brexipiprazole, NNH—number needed to harm, AE which affected >5% of participants are presented, *—as an adjunctive drug.

**Table 7:** The data on the adverse events of brexpiprazole vs. aripiprazole and cariprazine.

	EPS Other than Akathisia	Akathisia	Hyperprolactinemia	Preganany	Lactiation	Weight Increase and Metabolic Risk	Prolonged QTc Interval	Anticholinergic	Hepatotoxicity	Sedation	Hematologic Adverse Effects	Seizures	Hypotonia	Glaucoma
Aripiprazole	+	++/+++	0/−	++	++	0 *	0	0	0	0/+	+	0 ?	+	+ ?
Brexpiprazole	+	+	0/+ *	++	++	+/++	0 ?	0	0	+	0 ?	+	+	0/+ ?
Cariprazine	+	++/ +++	0 ?	?	+++	0/+	0	0	0/+	0	0 ?	0 ?	+	0/+ ?

+—increased risk, 0—no change in risk, −—decreased risk, ?—effect unknown, *—normalization of parameter was noted after switching to the drug.

**Table 8:** Values of the number needed to harm for the most important adverse events of brexpiprazole, cariprazine, lurasidone, and aripiprazole in patients with schizophrenia according to [69,70].

	Akathisia	Somnolence	Weight Increase
Aripiprazole	25	20	21
Brexipiprazole	112	50	17
Cariprazine	15	100	34
Lurasidone	11	20	43–150

## Data Availability

Not applicable.
